# Chiropractic students’ cognitive dissonance to statements about professional identity, role, setting and future: international perspectives from a secondary analysis of pooled data

**DOI:** 10.1186/s12998-021-00365-6

**Published:** 2021-02-02

**Authors:** Michael S. Swain, Jordan A. Gliedt, Katie de Luca, Dave Newell, Michelle Holmes

**Affiliations:** 1 Department of Chiropractic, Macquarie University, Balaclava Rd, Sydney, NSW 2109 Australia; 2 Chiropractic Academy of Research Leadership (CARL), Odense, Denmark; 3 Logan University College of Chiropractic, Chesterfield, USA; 4 Medical College of Wisconsin, Milwaukee, USA; 5 AECC University College, Bournemouth, UK

**Keywords:** Chiropractic, Students, health occupations, Education, professional, Cognitive dissonance, Interprofessional relations

## Abstract

**Background:**

Chiropractic students demonstrate philosophically opposing views about the chiropractic profession. The primary aim was to describe chiropractic students’ responses to statements about chiropractic identity, role, setting, and future direction. A secondary aim was to describe the frequency of internally conflicting responses.

**Methods:**

Three datasets from Europe, North America, and Australia/New Zealand were pooled in a secondary data analysis. Chiropractic students from 25 chiropractic training institutions completed interrelating surveys (combined response rate 21.9%) between 2013 and 2018. The survey instrument investigated student viewpoints about chiropractic professional identity, role, practice setting and future direction of chiropractic practice. Student attitudes about chiropractic were described using weighted proportions to adjust for unequal population sampling across the three geographical regions. The frequency of concordant and discordant student responses was described by combining identity items with items that explored responses about practice role, setting and future direction. The relationship between student characteristics (age, sex, education, association membership and geographical region) and ideologically conflicting responses were assessed using the Chi-squared test and Cramér’s V.

**Results:**

Data from 2396 student chiropractors (50.8% female; from Europe 36.2%, North America 49.6% and Australia/New Zealand 14.5%) were analysed. For identity, nearly half of the chiropractic students (weighted 45.1%) agreed that it is important for chiropractors to hold strongly to the traditional chiropractic theory that adjusting the spine corrects “dis-ease” and agreed (weighted 55.5%) that contemporary and evolving scientific evidence is more important than traditional chiropractic principles. The frequency of discordant (ideologically conflicting) student responses ranged from 32.5% for statements about identity versus role, to 51.4% for statements about identity versus future. There was no association between student age, sex and internally conflicting responses. Chiropractic students’ professional association membership status, pre-chiropractic education and geographical region were associated with ideologically conflicting responses.

**Conclusions:**

Chiropractic students in this analysis show traditional and progressive attitudes towards the chiropractic profession. Individual student responses frequently contradict in terms of professional ideology, but most (approximately half) students demonstrate concordant progressive and mainstream attitudes. Ideological conflict may raise concerns about some students’ ability to learn and make clinical judgements, and potential for disharmony in the chiropractic fraternity.

**Supplementary Information:**

The online version contains supplementary material available at 10.1186/s12998-021-00365-6.

## Background

Cognitive dissonance is exhibited when one possesses multiple paradoxical beliefs, or when an individual’s behaviour is inconsistent or conflicting with their beliefs [1]. The presence of cognitive dissonance generates an emotional reaction, prompting an individual to discard the source of conflict that can lead to overtly biased thinking and an increase in potentially conflicting behaviours [1]. The risk of experiencing cognitive dissonance may be higher when exposed to conflicting ideology amid divisive groups.

Chiropractic is a profession beleaguered with multiple groups asserting differing views of a predominant guiding ideological identity. The most documented contemporary ideological subgroups within the chiropractic profession can be broadly characterised by those who support a vitalistic philosophy with a focus on the ‘chiropractic vertebral subluxation’ and those who advocate for a science-based, biopsychosocial and musculoskeletal/spine focus [2, 3]. It is argued that detection and correction of ‘chiropractic vertebral subluxation’ is a separate and distinct paradigm [4] that provides chiropractic with a unique (vitalistic) identity [5]. Conversely, others argue that an ongoing devotion to vitalism is an impediment to providing best clinical care and inclusion in multi-disciplinary models of practice [6–8]. Proponents of the latter see movement towards a progressive identity centered around musculoskeletal spine care, evidence-based practice, and integration as necessary [9, 10]. Brosnan describes each approach as “*an attempt to diversify and expand the profession’s role: one* [science-based, musculoskeletal/spine focus] *by incorporating new approaches for a specific set of conditions; the other* [vitalistic] *by applying limited approaches to a wider range of conditions*” [2].

Chiropractic students may be subject to these differing viewpoints and may not fully comprehend the concepts and implications of a vitalistic and/or evidence-based paradigm. Recent regional studies have investigated chiropractic students’ ideology encompassing the profession’s identity and role [11, 12]. Results suggest the potential for cognitive dissonance in students, where internal conflicts in ideology are explained by a desire or an obligation to attempt to validate historical theories of the chiropractic profession [11, 12]. de Luca, et al. uncovered apparent internal conflicts among students from different chiropractic institutions in Australia and New Zealand, whereby the institution contributed most towards the explanation of a chiropractic student’s ideology [11].

This study expands upon prior investigations to further explore general identity issues facing chiropractic students internationally. Using survey data from Europe, North America, Australia and New Zealand our primary aim is to describe international chiropractic student opinions about chiropractic identity, role, setting, and future direction of clinical practice. The secondary aim is to describe the frequency of ideologically conflicting responses among chiropractic students.

## Methods

### Study design, setting, participants

This study was a secondary analysis of data from three primary studies conducted between 2013 and 2018 [11–13]. The primary studies were both paper and web-based cross-sectional surveys (combined response rate 21.9%) that explored chiropractic students’ views on chiropractic identity, role, setting and future professional practice. The surveys were conducted in Europe, North America, and Australia and New Zealand and included 822 (response rate 47.6%), 1247 (16.7% response rate), 347 (18.7% response rate) chiropractic students from 8, 12, and 5 institutions, respectively.

Ethical clearance or approval was sought and obtained from AECC University College Research Ethics Committee (2015), Logan University Institutional Review Board (Control # RD2023180530), and Human Research Ethics Committee of Macquarie University (Ref: 5201800259 & 5,201,830,413,414) prior to the commencement of this secondary analysis of existing data.

### Variables and measures

Data were collected using a survey instrument initially developed by Gliedt et al. [12]. Overlapping variables from the three primary studies were identified. Using three anonymised datasets, matching variables and data were merged into a single anonymised dataset for analysis.

Variables available for analysis were demographic characteristics and student viewpoints about chiropractic identity (2 ordinal Likert response items), role (2 ordinal Likert response items, 1 nominal response item), practice setting (2 ordinal Likert response items, 1 nominal response item) and future direction of chiropractic practice (2 ordinal Likert response items, 1 nominal response item). Demographic characteristics included (1) age, (2) sex, (3) education achieved prior to enrollment, (4) chiropractic association membership, and (5) geographical region.

Chiropractic students responded to 3 statements using nominal items concerning chiropractic role (1 = complementary/alternative health care practitioners, 2 = primary health care practitioners), setting (1 = integrated setting with other health-care disciplines, including general medicine, 2 = Integrated setting with alternative medicine practitioners only, 3 = alone or with other DCs without integration of any other health care discipline, 4 = any or all of the above) and future research priority (1 = physiological mechanisms of chiropractic adjustments, 2 = outcomes/cost-effectiveness of chiropractic care, 3 = outcomes/cost-effectiveness of integrated care models). Chiropractic students responded to 8 statements about chiropractic identity, setting, role and future using a 5-point Likert scale: 1 = strongly agree, 2 = agree, 3 = neutral, 4 = disagree, 5 = strongly disagree. To explore the nature of students' internal conflicts in ideology, responses to statements were categorised as either traditional/alternative or progressive/mainstream. Progressive/mainstream viewpoints were operationally defined as aligning with currently orthodox scientific views, whereas traditional/alternative viewpoints could be considered unorthodox to current evidence-based care and guidelines [14]. In order to categorise all respondents into a fourfold contingency table, Likert responses to statements were dichotomized, whereby: 1 = strongly agree and agree, and 2 = neutral, disagree, and strongly disagree. Based on the direction of the wording, responses to identity statements were classified as either 1 = traditional or 2 = progressive. Similarly, based on the direction of wording, responses to setting, role and future statements were classified as either 1 = alternative or 2 = mainstream. The frequency of concordant and discordant student opinions was described by combining identity items with items that explored opinions about practice role, setting and future direction, and coded as: 1 = ‘Concordant: Traditional & Alternative’, 2 = ‘Discordant: Progressive & Alternative’, 3 = ‘Discordant: Traditional & Mainstream’, or 4 = ‘Concordant: Progressive & Mainstream’.

### Statistical methods

Only participants with available data were included in the analyses. The characteristics of chiropractic students were summarised using descriptive statistics. Weightings based on respondent numbers were applied to all subsequent analyses to correct for unequal population sampling across the three geographical regions. The sample weights equally balanced student responses from Europe, North America, and Australia and New Zealand, the mean of the sample weights was equal to 1. Student responses to statements about chiropractic identity, setting, role and future as well as ideological concordant/discordant responses were described using weighted proportions and 95% confidence intervals (CI). To explain ideological concordance/discordance, we tested whether student characteristics (age, sex, education, association membership and geographical region) were associated with concordant/discordant responses using the Chi-squared test. The effect size of the associations was reported using Cramér’s V. Post-hoc validation analyses were conducted that compared the distribution of ideologically conflicting student responses, when ideological conflict was determined using a separate identity statement. All analyses were conducted using IBM SPSS Statistics for Windows, Version 25.0. Armonk, NY: IBM Corp. Graphical output of data were plotted using SigmaPlot version 12.0, Systat Software, Inc., San Jose California USA.

## Results

Data from a total of 2396 student chiropractors in 25 institutions across Europe, North America, Australia and New Zealand were available for analysis (Table 1).

**Table 1:** Characteristics of participants

	n	%
**Sex**		
Male	1172	49.2
Female	1212	50.8
**Age**		
16–25	1487	62.1
26–35	708	29.6
36–45	145	6.1
45–55	43	1.8
55+	10	0.4
**Area**		
Australia/New Zealand	347	14.5
Europe	867	36.2
North America	1182	49.3
**Highest level of education prior to enrolment**		
High school diploma	621	26.1
Bachelor degree	1309	55.0
Master degree	125	5.2
Doctoral degree	41	1.7
Other	277	11.6
**Membership of a Chiropractic Association**		
Yes	1087	45.5
No	1303	54.5

Table 2 reports the weighted relative frequency of student responses to statements about the identity, setting, role and future direction of the chiropractic profession. For identity, 76.5% (CI: 74.8–78.2%) of chiropractic students responded that chiropractors should be considered primary health care practitioners, and 23.5% (CI: 21.9–25.3%) complementary/alternative health care practitioners [*n* = 2381]. Students responded that the most appropriate setting for chiropractic care is integrated in a setting with other health-care disciplines, including general medicine (Table 3). Students responded that chiropractic researchers should focus their future efforts on physiological mechanisms of chiropractic adjustments 57.1% (CI: 55.1–59.1%), outcomes/cost-effectiveness of chiropractic care 27.6% (25.8–29.4%) and outcomes/cost-effectiveness of integrated care models 15.4% (CI: 13.9–16.8%) [*n* = 2361]. Additional file 1 - Supplementary table 1 reports the correlation between student responses to statements about identity, setting, role and future. Spearman’s rho coefficients ranged from positive r_s_ = 0.508, *P* < 0.01, to zero r_s_ = 0.0, *P* > 0.05, to negative r_s_ = − 0.478, P < 0.01 (see Additional file 1).

**Table 2:** Weighted relative frequency (95%CI) of participant responses to statements about chiropractic identity, setting, role and future

Identity	Strongly agree	Agree	Neutral	Disagree	Strongly disagree
1. It is important for chiropractors to strongly uphold the traditional chiropractic theory that adjusting the spine corrects “dis-ease” [*n* = 2323]	18.7% (17.1–20.3%) *Traditional*	26.4% (24.6–28.2%) *Traditional*	22.9% (21.2–24.6%) *Progressive*	19.4% (17.8–21.1%) *Progressive*	12.7% (11.4–14.1%) *Progressive*
2. Contemporary and evolving scientific evidence is more important than traditional chiropractic principles [*n* = 2377]	22.8% (21.2–24.5%) *Progressive*	32.7% (30.9–34.7%) *Progressive*	28.3% (26.5–30.1%) *Traditional*	13.1% (11.8–14.5%) *Traditional*	3.1% (2.4–3.8%) *Traditional*
**Setting**					
1. Inclusion of clinical chiropractic training internships and post-graduate positions in integrative medical settings are important to the progression of the chiropractic profession [*n* = 2384]	35% (33.1–36.9%) *Mainstream*	35.2% (33.3–37.2%) *Mainstream*	17.4% (16–19%) *Alternative*	7.6% (6.6–8.7%) *Alternative*	4.8% (4–5.7%) *Alternative*
2. Chiropractic providers should maintain its primary health care (direct access) status [*n* = 2381]	56.9% (54.9–58.9%)	31.1% (29.3–33%)	10.7% (9.5–11.9%)	0.9% (0.6–1.4%)	0.4% (0.2–0.7%)
**Role**					
1. Chiropractic intervention should consist of chiropractic adjustment only [*n* = 2390]	5.8% (4.9–6.8%) *Alternative*	10.1% (9–11.4%) *Alternative*	10.9% (9.7–12.2%) *Mainstream*	37.5% (35.6–39.4%) *Mainstream*	35.7% (33.8–37.6%) *Mainstream*
2. The chiropractic profession should expand its scope of practice to include prescription of medication, with appropriate advanced training [*n* = 2386]	9.8% (8.7–11%) *Mainstream*	16.7% (15.3–18.3%) *Mainstream*	17.2% (15.7–18.7%) *Alternative*	20.9% (19.3–22.6%) *Alternative*	35.4% (33.5–37.3%) *Alternative*
**Future**					
1. It is appropriate to allow for chiropractic theories to be updated and enhanced through the application and integration of current scientific advancements [*n* = 2388]	49.5% (47.5–51.5%) *Mainstream*	40.8% (38.8–42.8%) *Mainstream*	7.5% (6.5–8.6%) *Alternative*	1.5% (1.1–2.1%) *Alternative*	0.7% (0.4–1.1%) *Alternative*
2. It is appropriate for the chiropractic profession to distinguish and promote two separate subgroups of intervention. 1) Providing manual and other non-drug procedures 2) Providing subluxation correction only [*n* = 2389]	7.5% (6.5–8.6%) *Mainstream*	19% (17.4–20.6%) *Mainstream*	33.5% (31.6–35.4%) *Alternative*	24.7% (23–26.4%) *Alternative*	15.4% (14–16.9%) *Alternative*

The categorisation of statements as either traditional/alternative or progressive/mainstream are reported in *italics*

**Table 3:** Weighted relative frequency (95%CI) of responses to a statement about the most appropriate setting for chiropractic health care

	Integrated setting with other health-care disciplines, including general medicine	Integrated setting with alternative medicine practitioners only	Alone or with other DCs without integration of any other health care discipline	Any/All of the above
**Europe** **[*n* = 849]**	84.5% (81.7–86.8%)	7.3% (5.6–9.3%)	8.2% (6.4–10.3%)	
**North America** **[*n* = 1177]**	30.1% (26.9–33.3%)	4.3% (3–5.8%)	8.7% (6.9–10.8%)	56.9% (53.4–60.3%)
**Australia/New Zealand** **[*n* = 347]**	81.3% (78.4–83.8%)	5.2% (3.8–6.8%)	13.5% (11.3–16%)	

Table 4 reports the weighted relative frequency of conflicting student responses to statements about identity versus setting, role and future. Discordant (ideologically conflicting) student responses ranged from 32.5% (CI: 30.6–34.4%) for statements about identity versus role, to 51.4% (CI: 49.4–53.4%) for statements about identity versus future.

**Table 4:** Weighted relative frequency (95%CI) of conflicting responses to statements about identity versus setting, role and future

	Concordant: Traditional & Alternative	Discordant: Progressive & Alternative	Discordant: Traditional & Mainstream	Concordant: Progressive & Mainstream
**Identity (Traditional or Progressive)**	1. It is important for chiropractors to strongly uphold the traditional chiropractic theory that adjusting the spine corrects “dis-ease”			
**vs. Setting (Alternative or Mainstream)**				
1. Inclusion of clinical chiropractic training internships and post-graduate positions in integrative medical settings are important to the progression of the chiropractic profession [*n* = 2313]	19.9% (18.3–21.6%)	10.1% (8.9–11.4%)	25.1% (23.4–26.9%)	44.9% (42.9–46.9%)
2. Chiropractic providers should maintain its primary health care (direct access) status [*n* = 2309]	4.3% (3.5–5.2%)	7.6% (6.6–8.7%)	40.8% (38.8–42.8%)	47.3% (45.3–49.4%)
**vs. Role (Alternative or Mainstream)**				
1. Chiropractic intervention should consist of chiropractic adjustment only [*n* = 2317]	14.3% (12.9–15.8%)	1.8% (1.3–2.4%)	30.7% (28.9–32.6%)	53.2% (51.2–55.2%)
2. The chiropractic profession should expand its scope of practice to include prescription of medication, with appropriate advanced training [*n* = 2314]	39.5% (37.5–41.5%)	34.5% (32.6–36.5%)	5.6% (4.8–6.6%)	20.4% (18.8–22.1%)
**vs. Future (Alternative or Mainstream)**				
1. It is appropriate to allow for chiropractic theories to be updated and enhanced through the application and integration of current scientific advancements [*n* = 2317]	7.1% (6.1–8.2%)	2.3% (1.8–3%)	38% (36.1–40%)	52.6% (50.6–54.6%)
2. It is appropriate for the chiropractic profession to distinguish and promote two separate subgroups of intervention. 1) Providing manual and other non-drug procedures 2) Providing subluxation correction only [*n* = 2317]	33.5% (31.7–35.5%)	39.9% (37.9–41.9%)	11.5% (10.2–12.8%)	15% (13.6–16.6%)

**Fig. 1:**
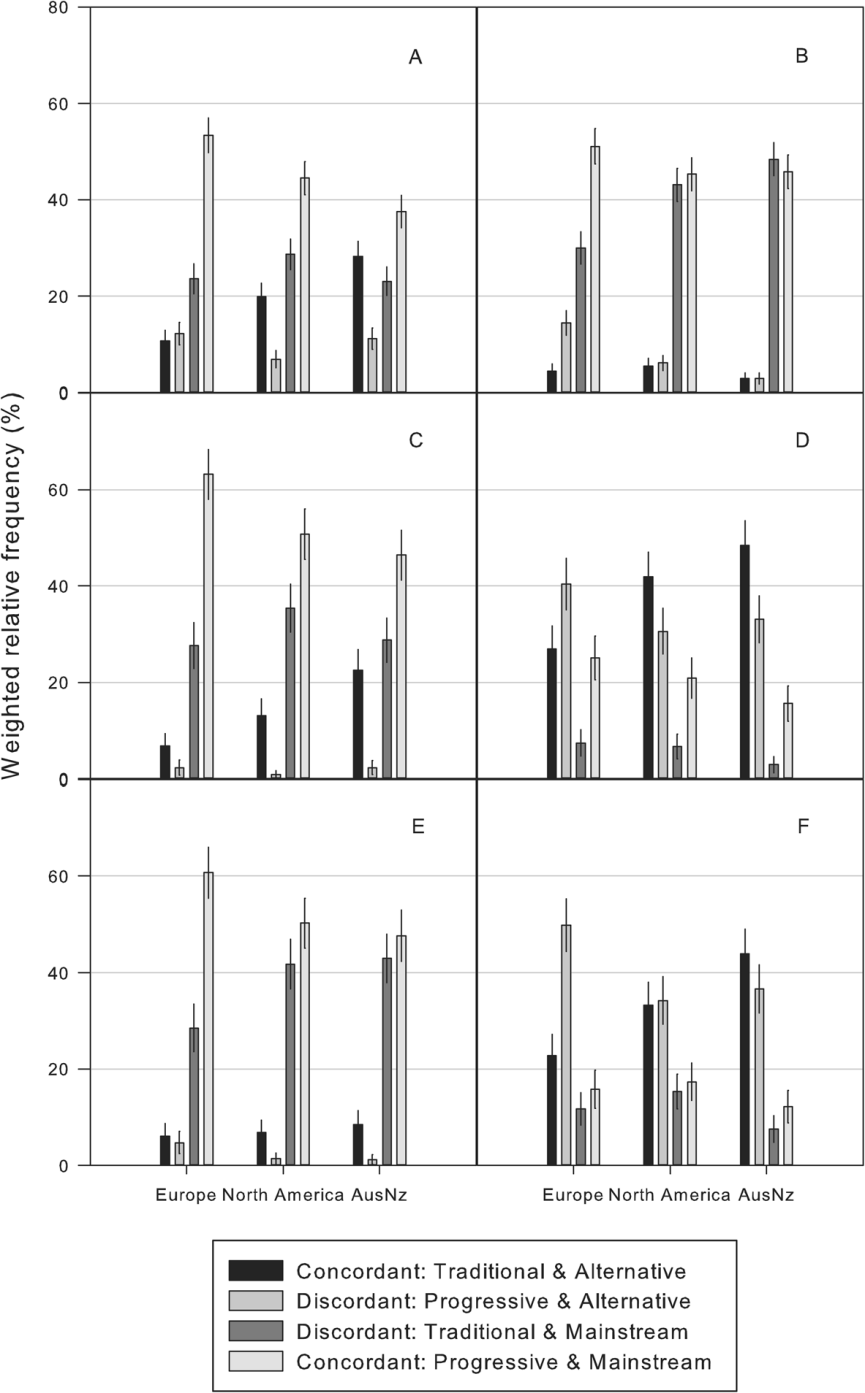
Weighted relative frequency (95%CI) of conflicting responses to statements about identity versus setting, role and future, by region. Key: **a** Identity versus Setting 1 (training); **b** Identity versus Setting 2 (primary care); **c** Identity versus Role 1 (adjustment); **d** Identity versus Role 2 (medication); **e** Identity versus Future 1 (theory); **f** Identity versus Future 2 (subgroups)

There was no relationship between student age, sex and ideologically conflicting student responses (see Additional file 1 - Supplementary tables: 2-3). Conversely, a chiropractic student’s professional association membership status, pre-chiropractic education and geographical region were associated with ideologically conflicting student responses (see Additional file 1 - Supplementary tables: 4-6). The strength of these associations were small (association membership status φ_c_ range: 0.06–0.07; df = 6 | pre-chiropractic education φ_c_ range: 0.09–0.12; df = 15) to moderate (geographical region φ_c_ range: 0.12–0.16, df = 6). Ideologically discordant responses were most pronounced in students from Europe for opinions about professional identity versus future direction (discordant: 61.5% [CI: 57.9–65%]), and least pronounced in students from Europe for opinions about professional identity versus the role of chiropractic in providing care (discordant: 30.0% [CI: 26.7–33.4%]). Figure 1 illustrates the frequency of conflicting responses reported to statements by geographical region.

**Fig. 2:**
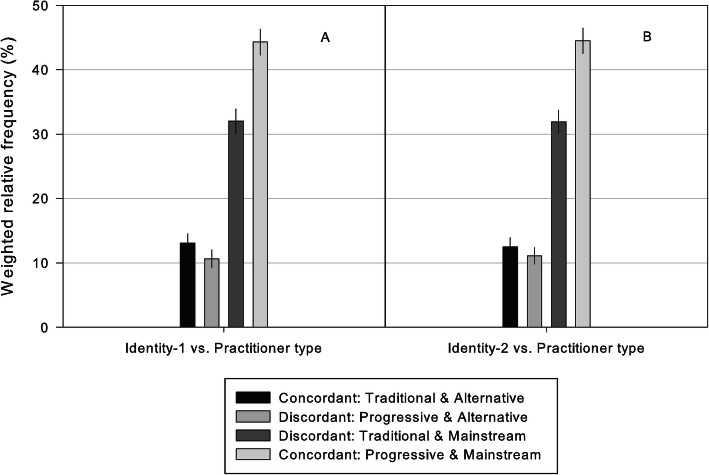
Weighted relative frequency (95%CI) of conflicting responses to statements about identity versus practitioner type. **Key:** Practitioner type statement: “Doctors of Chiropractic (DC) should be considered 1. complementary/alternative health care practitioners, or 2. primary health care practitioners”; **a** Identity statement 1 “It is important for chiropractors to strongly uphold the traditional chiropractic theory that adjusting the spine corrects ‘dis-ease’”; **b** Identity statement 2 “Contemporary and evolving scientific evidence is more important than traditional chiropractic principles”

Post-hoc validation analyses were conducted to compare the frequency distribution of conflicting responses to statements when cognitive dissonance was constructed using a separate identity statement. Figure 2 illustrates conflicting responses to identity versus “practitioner type” statements when internal conflict is constructed using two different identity statements. The rate of concordance and discordance between the two constructs of cognitive dissonance were strongly associated X^2^ = 2890.7, df = 9, *P* < 0.001; φ_c_ = 0.65. Using the ‘validation’ identity-2 statement, Additional file 1 - Supplementary table: 7 reports the weighted relative frequency of conflicting responses to statements about identity-2 versus setting, role and future.

## Discussion

Chiropractic students in this sample had both progressive and retrogressive ideological opinions about chiropractic professional practice. The patterns of conflicting responses were complex and occurred both within and across statements of identity, role, setting and future. The largest proportion of chiropractic students (up to approximately half) held concordant progressive and mainstream views. Albeit, reformist topics such as expanding scope-of-practice to include medication prescription and distinguishing ideologically different professional subgroups had a higher frequency of traditional/alternative and ideologically discordant opinions. Our analyses showed that geographical region may explain some of the variance around the frequency of students that report ideologically conflicting responses. Concordant progressive and mainstream views tended to be most frequent in European students and least frequent in Australian and New Zealand chiropractic students. European chiropractic students had the widest variation in frequency of dissonant perceptions. However, while the data presented here is useful for identifying response patterns within individuals, our study may not be generalisable and the frequencies of responses should be interpreted with caution.

Statements in the primary study surveys evaluated dimensions of professional attitude that were weak-to-moderately correlated across all students. Overall, our findings reflect other recent studies from North America [15] and Australia [16] that show chiropractic students currently hold professional attitudes on a continuum [17] from liberal/broad-scope (“interested in mixing elements of modern and alternative therapies into chiropractic practice”) to ‘conservative/straight/focused-scope’ (“chiropractors who believe in continuing the traditions of chiropractic to conservative”), but the majority fall somewhere between (middle-scope/mixer/pragmatic). A potential implication of this in the first instance, as described by Leboeuf-Yde et al., [18] is a significant, increasing and likely untenable disharmony within the chiropractic fraternity. Walker [8] extends the commentary to external implications, suggesting that the presence of retrograde ideologies cause the chiropractic profession reputational damage and act as barriers to legitimacy as a worthy and functioning allied health profession. The consequence of professional illegitimacy is thought to limit integration [advancement] in multi-disciplinary team-based care settings, [15, 19] reduce interprofessional collaboration [19] and fracture patient care experiences.

Our analysis is the first to directly explore the frequency and predictors of cognitive dissonance among chiropractic students, internationally. We estimate from one-third to over one-half of chiropractic students surveyed demonstrate some level of discordant professional opinion. Kline and McColl [1] highlight potentially serious implications of dissonance for healthcare students that includes defensiveness to negative feedback (i.e. an incapacity to critically reflect), hindered learning, erroneous self-awareness of clinical ability, and ultimately undermined clinical judgement (diagnostic error and outdated treatment approaches). The strongest predictors of dissonance among chiropractic students in our analyses are geographical region, and pre-chiropractic education. Our previous study [11] and additional contemporary research on student [15] and practitioner [20] professional identity shows that chiropractic (institution) programs explain much of the variance around professional identity and practice characteristics. This implies that educational stakeholders (institutions, faculty and accreditation bodies) may be responsible for much of the dissonance that hypothetically hinders learning and distorts clinical judgement. Gleberzon et al., further suggest the cognitive dissonance identified in our work [11, 12] could simply reflect the spectrum of diversity within the chiropractic profession to which students are being exposed [15]. We agree and suggest this may further imply some responsibility for student dissonance to professional associations, organisations and licensing/registration bodies that provide political leadership and develop and enforce standards for the chiropractic profession.

In our sample, Australian and New Zealand chiropractic students had the highest frequency of concordant traditional/alternative perceptions about chiropractic. Similarly, Innes et al., [16] recently conducted a study in Australia and concluded that non-evidence-based beliefs are common among Australian chiropractic students. The authors suggest that the chiropractic profession’s unique identity or ‘philosophy’ can be promoted as superior to science [16]. Preferment of theory may narrow chiropractic student views (act as a “cognitive lens”) to judge and reject the results from research evidence and guidelines. Potentially, cross-regional differences in the manner and extent ‘philosophy’ is promoted over science may explain some of the variance in chiropractic student dissonance identified in our analyses. Alternatively, cross-regional differences may be an artefact of response bias in so far as engaged students who respond to the survey may have different characteristics across institutions and regions.

Other educational confounders such as institutional lexicon might explain some of the variance around chiropractic student dissonance, not modelled in our study. An institution’s lexicon is the series of terms and narratives that reflect each chiropractic programs aspirations and ‘philosophy’ on the professional continuum [17]. Gleberzon et al., show differences between U.S. and Canadian chiropractic students by way of institutional lexicon and opinions on chiropractors role in healthcare [15]. Approximately 9% of Canadian vs. 86% of U.S. students are likely or very likely to use the term vertebral subluxation, whereas 46% of Canadian vs. 60% of U. S students consider chiropractic’s role in the healthcare system as ‘wellness-based’; it remains unclear if lexicon and role perceptions are linked [15]. Inherently linked to traditional chiropractic theories, the term ‘subluxation’ is found to varying extent in all but two U.S. chiropractic course catalogues and it’s use is significantly more common in U.S. than non-U.S institutions [21]. U.S. and Canadian accreditation standards still use the term ‘subluxation’, whereas there is no mention in the accreditation standards for Australasia or Europe [21]. Scholars have raised concerns over the use of the term ‘subluxation’ in teaching of students because: (1) the construct lacks sufficient scientific evidence and validity [22, 23] to be at all meaningful in health practitioner (chiropractic) training, and (2) using the term ‘subluxation’ adversely affects the clinical judgements of chiropractic students [24]. In the context of our analyses on cognitive dissonance, it may be that institutional lexicon is an important school-level predictor that varies by region. The implication is that a more hierarchical (multi-level) approach is necessary (i.e. students [level 1], nested within schools [level 2], nested within regions [level 3]) to explain the concept of cognitive dissonance among chiropractic students.

Different operating practices between Councils on Chiropractic Education (CCEs) (responsible for the accreditation of chiropractic institutions worldwide) may also explain some of the regional variation in student dissonance illustrated in our study. Innes et al., [19, 25–28] have comprehensively evaluated similarities and differences between the various CCEs in their content and prescription of accreditation standards. There are large differences between CCEs in the purpose of their mission statements, standards for faculty staff, requirements for clinical training by students, program budgetary autonomy and transparency, and curriculum [27]. At the most basic level, the educational standards and curriculum vary on subjects such as clinical decision making, chiropractic philosophy and history, research methods and procedures, practice ethics, practice management, and ‘wellness’, among several others. Innes et al., contends that the CCEs remain unclear in their directives to programs largely due to non-evidence-based input from stakeholders of ‘traditional’ chiropractic philosophy [27]. Qualitative interviews of nine CCE panel members further suggests the considerable variability between chiropractic programs worldwide is due to the embedded political negotiation process of CCEs determining their standards, whereby polite acceptance of ‘philosophical’ or ‘ideological’ views of some chiropractors persist [19]. The implication for the current research is that the frequency of student dissonance may endure until such time as patient care becomes the highest priority in the CCE’s formulation and prescription of accreditation standards.

Curiosity about apparent internal conflict among chiropractic students arose from our previous research and led us to design this secondary analysis. A strength of our question driven approach is the availability of existing data and specific variables needed to address the research questions of interest. In this study we have pooled three matching datasets to obtain an international perspective on chiropractic students. By applying sample weights to rebalance the data we have obtained improved accuracy and representability in our estimates of student opinion. Our previous research points towards profession related cognitive dissonance within groups of chiropractic students. Our current analysis uniquely uses the multiple constructs of past survey items to describe the multidimensional nature and frequency of paradoxical responses among chiropractic students. The major limitations of our approach relate to the cross-sectional nature of the primary survey research. The primary surveys all had low rates of recruitment; sampling bias likely undermines the external validity of our estimates and the generalisability of our findings. It is unclear if student preparedness to answer questions about chiropractic professional attitudes, or some other aspect of student engagement explains the low response rate. The three surveys were collected at different periods in time and although sampling weights account for regional differences some temporal drift may be present in our estimates. Some of our analyses observed a statistical association between independent and dependent variables. Our analyses do not consider temporal sequencing of events, or confounding, and cannot infer causal effects. Finally, the measurement properties of items used to evaluate the construct of chiropractic ideology remain unevaluated; observational error may be a source of bias in our estimates.

There is urgent need for further research to describe what chiropractic is, [29] in order to improve student learning and teaching, develop professional scholarship and penultimately improve patient care. Future studies must emphasise student engagement, potentially through incentivisation to improve research participation. Research from North America [20] and Europe [30] shows that a chiropractor’s identity influences their clinical practice characteristics. While it is well accepted that various cognitive biases can affect clinical decision-making, future research is needed to understand the impacts, if any, of chiropractic ideological conflict on the outcomes of students, chiropractors, and the patients that they serve. Several researchers have attempted to measure the conceptual elements of chiropractic ‘philosophy’ [12, 14, 15, 31]. Biggs et al., point out that these measurement tools are a touchstone for a myriad of complex issues that require identification and operationalisation [31]. Further research is needed to improve the understanding of measurement properties of tools used in research and to aid their selection and use.

## Conclusions

Chiropractic students in this study show both traditional and progressive attitudes towards the identity, role, setting and future of the chiropractic profession. In some students, perceptions on chiropractic identity, role, setting and future contradict in ideology, which we attribute to cognitive dissonance. This raises several hypothetical concerns including some students impaired ability to learn and make clinical judgements, potential for disharmony in the chiropractic fraternity, an illegitimacy amongst other healthcare professions and organisations. Educational stakeholders would be prudent to deliver clear and consistent curricula that are integrable across international chiropractic programs and relatable to other health disciplines. Future research is needed to better engage students and improve the operationalisation of chiropractic ‘philosophy’, so that the complex impacts on students, chiropractors and patients can be understood.

## Supplementary Information

**Additional file 1: Table S1.** Spearman’s rho correlation coefficients reporting the relationship between identity, setting, role and future responses. **Table S2.** Weighted relative frequency (95%CI) of conflicting responses to statements about identity versus setting, role and future, by sex. **Table S3.** Weighted relative frequency (95%CI) of conflicting responses to statements about identity versus setting, role and future, by age-group. **Table S4.** Weighted relative frequency (95%CI) of conflicting responses to statements about identity versus setting, role and future, by region. **Table S5.** Weighted relative frequency (95%CI) of conflicting responses to statements about identity versus setting, role and future, by pre-enrolment education **. Table S6.** Weighted relative frequency (95%CI) of conflicting responses to statements about identity versus setting, role and future, by association membership **. Table S7.** Weighted relative frequency (95%CI) of conflicting responses to statements about identity-2 versus setting, role and future

## Data Availability

The dataset used during the current study are available from the corresponding author on reasonable request.
