# Supplementary material for: Chiropractic students’ cognitive dissonance to statements about professional identity, role, setting and future: international perspectives from a secondary analysis of pooled data
Source: Chiropr Man Therap. 2021 Feb 2;29:5. doi: 10.1186/s12998-021-00365-6 (PMC7851948; doi:10.1186/s12998-021-00365-6)
Supplement: Supplementary file 1 — Additional file 1: Table S1. Spearman’s rho correlation coefficients reporting the relationship between identity, setting, role and future responses. Table S2. Weighted relative frequency (95%CI) of conflicting responses to statements about identity versus setting, role and future, by sex. Table S3. Weighted relative frequency (95%CI) of conflicting responses to statements about identity versus setting, role and future, by age-group. Table S4. Weighted relative frequency (95%CI) of conflicting responses to statements about identity versus setting, role and future, by region. Table S5. Weighted relative frequency (95%CI) of conflicting responses to statements about identity versus setting, role and future, by pre-enrolment education . Table S6. Weighted relative frequency (95%CI) of conflicting responses to statements about identity versus setting, role and future, by association membership . Table S7. Weighted relative frequency (95%CI) of conflicting responses to statements about identity-2 versus setting, role and future [file 12998_2021_365_MOESM1_ESM.docx]

**Supplementary table 1.** Spearman's rho coefficients reporting the correlation between identity, setting, role and future responses.

|  | **Identity 1** | **Identity 2** | **Setting 1** | **Setting 2** | **Role 1** | **Role 2** | **Future 1** | **Future 2** |
| --- | --- | --- | --- | --- | --- | --- | --- | --- |
| **Identity** |  |  |  |  |  |  |  |  |
| 1. It is important for chiropractors to strongly uphold the traditional chiropractic theory that adjusting the spine corrects "dis-ease" | 1 | -.478** | -.322** | .066** | .508** | -.443** | -.304** | -.058** |
| 2. Contemporary and evolving scientific evidence is more important than traditional chiropractic principles | -.478** | 1 | .339** | -0.001 | -.350** | .371** | .466** | .076** |
| **Setting** |  |  |  |  |  |  |  |  |
| 1. Inclusion of clinical chiropractic training internships and post-graduate positions in integrative medical settings are important to the progression of the chiropractic profession | -.322** | .339** | 1 | .151** | -.392** | .392** | .321** | .106** |
| 2. Chiropractic providers should maintain its primary health care (direct access) status | .066** | -0.001 | .151** | 1 | -.047* | -.098** | .162** | -.080** |
| **Role** |  |  |  |  |  |  |  |  |
| 1. Chiropractic intervention should consist of chiropractic adjustment only | .508** | -.350** | -.392** | -.047* | 1 | -.368** | -.293** | -0.027 |
| 2. The chiropractic profession should expand its scope of practice to include prescription of medication, with appropriate advanced training | -.443** | .371** | .392** | -.098** | -.368** | 1 | .230** | .142** |
| **Future** |  |  |  |  |  |  |  |  |
| 1. It is appropriate to allow for chiropractic theories to be updated and enhanced through the application and integration of current scientific advancements | -.304** | .466** | .321** | .162** | -.293** | .230** | 1 | 0.018 |
| 2. It is appropriate for the chiropractic profession to distinguish and promote two separate subgroups of intervention. 1) Providing manual and other non-drug procedures 2) Providing subluxation correction only | -.058** | .076** | .106** | -.080** | -0.027 | .142** | 0.018 | 1 |

** Correlation is significant at the 0.01 level (2-tailed).

* Correlation is significant at the 0.05 level (2-tailed).

**Supplementary table 2.** Weighted relative frequency (95%CI) of conflicting responses to statements about identity versus setting, role and future, by sex

|  | **n** | **Concordant: Traditional & Alternative** | **Discordant: Progressive & Alternative** | **Discordant: Traditional & Mainstream** | **Concordant: Progressive & Mainstream** | **Chi-Square Tests  Cramér's V** |
| --- | --- | --- | --- | --- | --- | --- |
| **Internal conflict: Identity vs. Setting (Training)** | | | | | | |
| Male | 1141 | 21.4% (19.1%-23.9%) | 10.2% (8.5%-12.1%) | 23.5% (21.1%-26.1%) | 44.9% (42.1%-47.9%) | X^2^=4.668, df=3, P=0.198  Cramér's V=0.045 |
| Female | 1161 | 18.5% (16.4%-20.8%) | 10% (8.4%-11.8%) | 26.5% (24.1%-29.1%) | 45% (42.2%-47.8%) |  |
| **Internal conflict: Identity vs. Setting (Primary care)** | | | | | | |
| Male | 1138 | 4.7% (3.5%-6%) | 7.3% (5.9%-9%) | 40.3% (37.4%-43.2%) | 47.7% (44.8%-50.6%) | X^2^=1.074, df=3, P=0.783  Cramér's V=0.022 |
| Female | 1160 | 3.9% (2.9%-5.2%) | 7.9% (6.5%-9.5%) | 41.2% (38.4%-44%) | 47% (44.2%-49.9%) |  |
| **Internal conflict: Identity vs. Role (Adjustment)** | | | | | | |
| Male | 1143 | 15.3% (13.3%-17.5%) | 1.7% (1.1%-2.6%) | 29.6% (27%-32.3%) | 53.4% (50.5%-56.3%) | X^2^=2.133, df=3, P=0.545  Cramér's V=0.03 |
| Female | 1162 | 13.5% (11.6%-15.5%) | 1.8% (1.1%-2.6%) | 31.6% (29%-34.2%) | 53.1% (50.3%-56%) |  |
| **Internal conflict: Identity vs. Role (Medication)** | | | | | | |
| Male | 1143 | 39.9% (37%-42.8%) | 32.7% (30%-35.5%) | 5.1% (3.9%-6.5%) | 22.4% (20%-24.9%) | X^2^=7.279, df=3, P=0.064  Cramér's V=0.056 |
| Female | 1159 | 39% (36.3%-41.8%) | 36.3% (33.5%-39%) | 6.1% (4.9%-7.6%) | 18.6% (16.5%-20.9%) |  |
| **Internal conflict: Identity vs. Future (Theory)** | | | | | | |
| Male | 1144 | 7.3% (5.8%-8.8%) | 2.6% (1.8%-3.6%) | 37.7% (34.9%-40.6%) | 52.5% (49.6%-55.4%) | X^2^=0.658, df=3, P=0.883  Cramér's V=0.017 |
| Female | 1161 | 7% (5.7%-8.6%) | 2.1% (1.4%-3%) | 38.1% (35.4%-40.9%) | 52.8% (50%-55.6%) |  |
| **Internal conflict: Identity vs. Future (Subgroups)** | | | | | | |
| Male | 1143 | 33.3% (30.6%-36.1%) | 39.1% (36.2%-42%) | 11.7% (9.9%-13.7%) | 16% (13.9%-18.2%) | X^2^=1.854, df=3, P=0.603  Cramér's V=0.028 |
| Female | 1162 | 33.8% (31.2%-36.6%) | 40.8% (38%-43.6%) | 11.2% (9.5%-13.1%) | 14.2% (12.3%-16.2%) |  |

**Supplementary table 3.** Weighted relative frequency (95%CI) of conflicting responses to statements about identity versus setting, role and future, by age-group

|  | **n** | **Concordant: Traditional & Alternative** | **Discordant: Progressive & Alternative** | **Discordant: Traditional & Mainstream** | **Concordant: Progressive & Mainstream** | **Chi-Square Tests  Cramér's V** |
| --- | --- | --- | --- | --- | --- | --- |
| **Internal conflict: Identity vs. Setting (Training)** | | | | | | |
| 16-25 years | 1415 | 19.8% (17.9%-22%) | 10.6% (9.1%-12.3%) | 24% (21.9%-26.3%) | 45.5% (43%-48.1%) | X^2^=12.325, df=12, P=0.42  Cramér's V=0.042 |
| 26-35 years | 699 | 20.2% (17.2%-23.4%) | 8.3% (6.4%-10.7%) | 28.1% (24.7%-31.7%) | 43.4% (39.5%-47.2%) |  |
| 36-45 years | 144 | 21.7% (15.7%-28.8%) | 13.7% (9%-20%) | 22.1% (16.3%-29.5%) | 42.5% (35.1%-50.7%) |  |
| 46-55 years | 43 | 14.5% (6.5%-25.5%) | 6.3% (1.7%-15.2%) | 27.8% (17%-41.4%) | 51.4% (38.4%-65.4%) |  |
| > 55 years | 10 | 5.7% (0.9%-32.8%) | 11.4% (0.9%-32.8%) | 32.8% (12.5%-61.2%) | 50.1% (24.3%-75.7%) |  |
| **Internal conflict: Identity vs. Setting (Primary care)** | | | | | | |
| 16-25 years | 1414 | 4.3% (3.4%-5.4%) | 8.7% (7.3%-10.2%) | 39.6% (37.2%-42.2%) | 47.4% (44.8%-50%) | X^2^=17.786, df=12, P=0.122  Cramér's V=0.051 |
| 26-35 years | 699 | 4.4% (3%-6.1%) | 6.1% (4.4%-8.1%) | 43.9% (40%-47.6%) | 45.6% (41.8%-49.5%) |  |
| 36-45 years | 143 | 1.8% (0.6%-5.2%) | 6.2% (3%-10.5%) | 41.8% (33.8%-49.4%) | 50.2% (42.1%-57.9%) |  |
| 46-55 years | 42 | 9.2% (4%-20.9%) | 1.9% (0.2%-9.1%) | 33.8% (22.5%-48.6%) | 55.1% (41.2%-68.4%) |  |
| > 55 years | 10 | 5.7% (0.9%-32.8%) | 0% | 32.8% (12.5%-61.2%) | 61.5% (31.2%-82%) |  |
| **Internal conflict: Identity vs. Role (Adjustment)** | | | | | | |
| 16-25 years | 1416 | 14.2% (12.5%-16.1%) | 1.5% (1%-2.2%) | 29.6% (27.3%-32%) | 54.6% (52%-57.1%) | X^2^=17.257, df=12, P=0.14  Cramér's V=0.05 |
| 26-35 years | 702 | 16.6% (13.8%-19.6%) | 2.3% (1.4%-3.7%) | 31.7% (28.2%-35.4%) | 49.4% (45.5%-53.2%) |  |
| 36-45 years | 144 | 7.9% (4.4%-13%) | 2.6% (0.9%-6.1%) | 35.9% (28.9%-44%) | 53.6% (46%-61.7%) |  |
| 46-55 years | 43 | 11.2% (5.2%-23.1%) | 0% | 31% (20.4%-45.7%) | 57.8% (44.2%-70.9%) |  |
| > 55 years | 10 | 0% | 0% | 38.5% (18%-68.8%) | 61.5% (31.2%-82%) |  |
| **Internal conflict: Identity vs. Role (Medication)** | | | | | | |
| 16-25 years | 1418 | 38.6% (36.1%-41.1%) | 35.8% (33.4%-38.3%) | 5.3% (4.3%-6.6%) | 20.3% (18.3%-22.4%) | X^2^=18.401, df=12, P=0.104  Cramér's V=0.051 |
| 26-35 years | 700 | 41.8% (38.1%-45.7%) | 30.5% (27.1%-34.2%) | 6.6% (4.8%-8.6%) | 21.1% (18.1%-24.4%) |  |
| 36-45 years | 142 | 39.5% (32.2%-47.7%) | 40.2% (32.8%-48.3%) | 4.2% (1.7%-8%) | 16.1% (10.7%-22.3%) |  |
| 46-55 years | 42 | 35.6% (24.3%-50.7%) | 37.1% (24.3%-50.7%) | 7.4% (2.8%-18.2%) | 19.9% (11%-33.2%) |  |
| > 55 years | 10 | 32.8% (12.5%-61.2%) | 11.4% (0.9%-32.8%) | 5.7% (0.9%-32.8%) | 50.1% (24.3%-75.7%) |  |
| **Internal conflict: Identity vs. Future (Theory)** | | | | | | |
| 16-25 years | 1419 | 7.2% (5.9%-8.6%) | 2.8% (2%-3.7%) | 36.7% (34.3%-39.2%) | 53.2% (50.7%-55.8%) | X^2^=12.941, df=12, P=0.373  Cramér's V=0.043 |
| 26-35 years | 699 | 7.3% (5.5%-9.5%) | 1.6% (0.8%-2.7%) | 41% (37.3%-44.9%) | 50.1% (46.2%-53.9%) |  |
| 36-45 years | 144 | 4.8% (2.1%-8.8%) | 1.7% (0.6%-5.2%) | 39.1% (32%-47.4%) | 54.5% (46.7%-62.4%) |  |
| 46-55 years | 43 | 5.9% (1.7%-15.2%) | 0% | 36.3% (23.8%-49.8%) | 57.8% (44.2%-70.9%) |  |
| > 55 years | 10 | 19.4% (3.6%-43.6%) | 0% | 19.1% (3.6%-43.6%) | 61.5% (31.2%-82%) |  |
| **Internal conflict: Identity vs. Future (Subgroups)** | | | | | | |
| 16-25 years | 1416 | 32.8% (30.5%-35.3%) | 40% (37.5%-42.5%) | 11.1% (9.5%-12.8%) | 16.1% (14.3%-18.1%) | X^2^=15.929, df=12, P=0.195  Cramér's V=0.048 |
| 26-35 years | 702 | 35.6% (32%-39.4%) | 37.4% (33.8%-41.2%) | 12.6% (10.3%-15.4%) | 14.3% (11.7%-17.1%) |  |
| 36-45 years | 144 | 34.6% (27.6%-42.7%) | 46.4% (38.9%-54.6%) | 9.3% (5.4%-14.6%) | 9.7% (5.9%-15.4%) |  |
| 46-55 years | 43 | 24% (13.8%-37.1%) | 49.2% (36.5%-63.5%) | 18.2% (9.3%-30.3%) | 8.6% (2.8%-17.9%) |  |
| > 55 years | 10 | 32.8% (12.5%-61.2%) | 44.4% (18%-68.8%) | 5.7% (0.9%-32.8%) | 17.1% (3.6%-43.6%) |  |

**Supplementary table 4.** Weighted relative frequency (95%CI) of conflicting responses to statements about identity versus setting, role and future, by region

|  | **n** | **Concordant: Traditional & Alternative** | **Discordant: Progressive & Alternative** | **Discordant: Traditional & Mainstream** | **Concordant: Progressive & Mainstream** | **Chi-Square Tests  Cramér's V** |
| --- | --- | --- | --- | --- | --- | --- |
| **Internal conflict: Identity vs. Setting (Training)** | | | | | | |
| Europe | 792 | 10.7% (8.6%-13.1%) | 12.2% (10%-14.7%) | 23.6% (20.6%-26.8%) | 53.4% (49.9%-57.1%) | X^2^=98.604, df=6, P<0.001  Cramér's V=0.146. |
| North America | 1174 | 19.9% (17.2%-22.8%) | 6.9% (5.3%-8.9%) | 28.6% (25.5%-31.8%) | 44.5% (41.2%-48.1%) |  |
| AusNz | 347 | 28.2% (25.2%-31.4%) | 11.2% (9.2%-13.6%) | 23.1% (20.2%-26.1%) | 37.5% (34.2%-40.9%) |  |
| **Internal conflict: Identity vs. Setting (Primary care)** | | | | | | |
| Europe | 790 | 4.4% (3.1%-6.1%) | 14.4% (12%-17.1%) | 30% (26.7%-33.4%) | 51.1% (47.5%-54.8%) | X^2^=113.701, df=6, P<0.001  Cramér's V=0.157. |
| North America | 1172 | 5.5% (4.1%-7.3%) | 6.1% (4.6%-7.9%) | 43.1% (39.6%-46.5%) | 45.3% (41.9%-48.8%) |  |
| AusNz | 347 | 2.9% (1.9%-4.2%) | 2.9% (1.9%-4.2%) | 48.4% (44.9%-51.8%) | 45.8% (42.4%-49.3%) |  |
| **Internal conflict: Identity vs. Role (Adjustment)** | | | | | | |
| Europe | 794 | 6.8% (5.2%-8.8%) | 2.3% (1.4%-3.6%) | 27.7% (24.6%-31.1%) | 63.2% (59.7%-66.6%) | X^2^=103.164, df=6, P<0.001  Cramér's V=0.149. |
| North America | 1176 | 13.1% (10.9%-15.6%) | 0.8% (0.3%-1.5%) | 35.4% (32.1%-38.7%) | 50.8% (47.3%-54.3%) |  |
| AusNz | 347 | 22.5% (19.6%-25.4%) | 2.3% (1.4%-3.5%) | 28.8% (25.8%-32%) | 46.4% (42.9%-49.8%) |  |
| **Internal conflict: Identity vs. Role (Medication)** | | | | | | |
| Europe | 792 | 27% (23.9%-30.3%) | 40.4% (36.9%-44.1%) | 7.4% (5.7%-9.5%) | 25.1% (22.1%-28.3%) | X^2^=91.035, df=6, P<0.001  Cramér's V=0.14. |
| North America | 1175 | 41.9% (38.5%-45.4%) | 30.6% (27.5%-33.9%) | 6.7% (5.1%-8.6%) | 20.9% (18.2%-23.8%) |  |
| AusNz | 347 | 48.4% (44.9%-51.8%) | 33.1% (29.9%-36.4%) | 2.9% (1.9%-4.2%) | 15.6% (13.2%-18.2%) |  |
| **Internal conflict: Identity vs. Future (Theory)** | | | | | | |
| Europe | 792 | 6.1% (4.5%-7.9%) | 4.7% (3.3%-6.4%) | 28.5% (25.3%-31.9%) | 60.7% (57.2%-64.3%) | X^2^=67.622, df=6, P<0.001  Cramér's V=0.121. |
| North America | 1178 | 6.8% (5.2%-8.7%) | 1.4% (0.7%-2.4%) | 41.7% (38.3%-45.2%) | 50.2% (46.8%-53.7%) |  |
| AusNz | 347 | 8.4% (6.6%-10.5%) | 1.2% (0.6%-2%) | 42.9% (39.6%-46.4%) | 47.6% (44%-51%) |  |
| **Internal conflict: Identity vs. Future (Subgroups)** | | | | | | |
| Europe | 793 | 22.7% (19.8%-25.9%) | 49.8% (46.2%-53.5%) | 11.7% (9.6%-14.3%) | 15.8% (13.2%-18.5%) | X^2^=105.605, df=6, P<0.001  Cramér's V=0.151. |
| North America | 1177 | 33.2% (30%-36.5%) | 34.2% (30.9%-37.5%) | 15.3% (13%-18%) | 17.3% (14.8%-20.1%) |  |
| AusNz | 347 | 43.8% (40.3%-47.2%) | 36.6% (33.3%-40%) | 7.5% (5.8%-9.5%) | 12.1% (10%-14.6%) |  |

**Supplementary table 5.** Weighted relative frequency (95%CI) of conflicting responses to statements about identity versus setting, role and future, by pre-enrolment education

|  | **n** | **Concordant: Traditional & Alternative** | **Discordant: Progressive & Alternative** | **Discordant: Traditional & Mainstream** | **Concordant: Progressive & Mainstream** | **Chi-Square Tests  Cramér's V** |
| --- | --- | --- | --- | --- | --- | --- |
| **Internal conflict: Identity vs. Setting (Training)** | | | | | | |
| High school diploma | 579 | 15.9% (13.4%-18.7%) | 13.7% (11.3%-16.3%) | 23.4% (20.4%-26.5%) | 47% (43.3%-50.5%) | X^2^=87.662, df=15, P<0.001 Cramér's V=0.112 |
| Bachelor degree (BSc, BA, etc) | 1282 | 18.7% (16.5%-21.1%) | 7.4% (6%-9.1%) | 25.9% (23.3%-28.6%) | 48% (45%-51%) |  |
| Master degree (MSc, MA, etc) | 122 | 12.4% (7.4%-19.7%) | 11.2% (6%-17.6%) | 24.5% (17.1%-32.9%) | 51.9% (42.1%-60.5%) |  |
| Doctoral degree (PhD, EdD, etc.) | 38 | 15.2% (6.2%-30.9%) | 5.1% (1.3%-18.6%) | 25.3% (12.6%-41.7%) | 54.5% (36.2%-69.5%) |  |
| Other | 272 | 33.8% (29.1%-38.8%) | 10.7% (7.9%-14.3%) | 26.2% (22%-31%) | 29.3% (24.8%-34.2%) |  |
| Missing | 7 | 14.3% (1.9%-55.8%) | 14.3% (1.9%-55.8%) | 28.6% (7.7%-71.4%) | 42.9% (16.7%-83.3%) |  |
| **Internal conflict: Identity vs. Setting (Primary care)** | | | | | | |
| High school diploma | 578 | 3.9% (2.7%-5.5%) | 9.7% (7.6%-11.9%) | 35.4% (32.1%-39%) | 51.1% (47.4%-54.6%) | X^2^=55.083, df=15, P<0.001 Cramér's V=0.089 |
| Bachelor degree (BSc, BA, etc) | 1279 | 4.6% (3.5%-6%) | 6.9% (5.5%-8.5%) | 40% (37.1%-43%) | 48.5% (45.5%-51.5%) |  |
| Master degree (MSc, MA, etc) | 121 | 2.3% (0.8%-7.1%) | 8.3% (4.1%-14.4%) | 34.9% (26.2%-43.7%) | 54.5% (45.2%-63.6%) |  |
| Doctoral degree (PhD, EdD, etc.) | 39 | 7.8% (2.7%-23%) | 7.8% (2.7%-23%) | 34.4% (19.8%-51.6%) | 50.1% (33.3%-66.7%) |  |
| Other | 271 | 4.2% (2.5%-6.6%) | 5.3% (3.3%-8%) | 55.9% (50.7%-60.9%) | 34.6% (29.9%-39.7%) |  |
| Missing | 7 | 14.3% (1.9%-55.8%) | 14.3% (1.9%-55.8%) | 28.6% (7.7%-71.4%) | 42.9% (16.7%-83.3%) |  |
| **Internal conflict: Identity vs. Role (Adjustment)** | | | | | | |
| High school diploma | 580 | 10.9% (8.8%-13.3%) | 1.6% (0.9%-2.7%) | 28.6% (25.4%-31.9%) | 59% (55.4%-62.5%) | X^2^=98.973, df=15, P<0.001 Cramér's V=0.119 |
| Bachelor degree (BSc, BA, etc) | 1282 | 12.6% (10.7%-14.7%) | 1.7% (1%-2.6%) | 31.9% (29.2%-34.8%) | 53.8% (50.8%-56.8%) |  |
| Master degree (MSc, MA, etc) | 122 | 9.9% (5.4%-16.5%) | 2.1% (0.4%-5.7%) | 27% (19.4%-35.8%) | 61% (52%-69.9%) |  |
| Doctoral degree (PhD, EdD, etc.) | 39 | 0% | 2.8% (0.3%-13.7%) | 42.2% (27.7%-60.9%) | 55% (39.1%-72.3%) |  |
| Other | 273 | 28.9% (24.3%-33.5%) | 2% (0.9%-3.8%) | 31% (26.3%-35.8%) | 38.2% (33.2%-43.2%) |  |
| Missing | 7 | 14.3% (1.9%-55.8%) | 14.3% (1.9%-55.8%) | 28.6% (7.7%-71.4%) | 42.9% (16.7%-83.3%) |  |
| **Internal conflict: Identity vs. Role (Medication)** | | | | | | |
| High school diploma | 578 | 33.6% (30.2%-37%) | 39.6% (36.2%-43.2%) | 5.9% (4.3%-7.7%) | 21% (18.2%-24%) | X^2^=66.723, df=15, P<0.001 Cramér's V=0.098 |
| Bachelor degree (BSc, BA, etc) | 1282 | 39.2% (36.3%-42.1%) | 33.9% (31.2%-36.9%) | 5.4% (4.1%-6.8%) | 21.5% (19.1%-24%) |  |
| Master degree (MSc, MA, etc) | 121 | 27.8% (20.4%-37.1%) | 43% (33.8%-52.1%) | 9.3% (4.8%-15.5%) | 19.8% (13.4%-28.2%) |  |
| Doctoral degree (PhD, EdD, etc.) | 39 | 31% (17.3%-48.4%) | 30.4% (17.3%-48.4%) | 11.2% (4.4%-27%) | 27.4% (14.9%-45.1%) |  |
| Other | 273 | 55.9% (50.7%-60.9%) | 24.5% (20.4%-29.2%) | 4% (2.2%-6.2%) | 15.7% (12.3%-19.8%) |  |
| Missing | 7 | 42.9% (16.7%-83.3%) | 42.9% (16.7%-83.3%) | 0% | 14.3% (1.9%-55.8%) |  |
| **Internal conflict: Identity vs. Future (Theory)** | | | | | | |
| High school diploma | 579 | 6.3% (4.8%-8.3%) | 2.9% (1.9%-4.4%) | 33% (29.7%-36.4%) | 57.7% (54.1%-61.2%) | X^2^=54.013, df=15, P<0.001 Cramér's V=0.088 |
| Bachelor degree (BSc, BA, etc) | 1284 | 7% (5.5%-8.6%) | 2% (1.3%-3.1%) | 37.6% (34.7%-40.5%) | 53.4% (50.4%-56.4%) |  |
| Master degree (MSc, MA, etc) | 122 | 6% (2.9%-12%) | 0.8% (0.1%-4.1%) | 30.9% (22.6%-39.6%) | 62.3% (52.9%-70.8%) |  |
| Doctoral degree (PhD, EdD, etc.) | 38 | 5.8% (1.3%-18.6%) | 2.9% (0.3%-13.7%) | 34.6% (19.8%-51.6%) | 56.6% (39.1%-72.3%) |  |
| Other | 272 | 9.1% (6.5%-12.4%) | 2.5% (1.2%-4.5%) | 50.9% (45.8%-56.1%) | 37.5% (32.5%-42.5%) |  |
| Missing | 8 | 25% (6.5%-64.8%) | 0% | 25% (6.5%-64.8%) | 50% (23.5%-86.1%) |  |
| **Internal conflict: Identity vs. Future (Subgroups)** | | | | | | |
| High school diploma | 580 | 29.4% (26.2%-32.8%) | 43.5% (40.1%-47.2%) | 10% (8%-12.3%) | 17% (14.4%-19.8%) | X^2^=69.64, df=15, P<0.001 Cramér's V=0.1 |
| Bachelor degree (BSc, BA, etc) | 1283 | 31.9% (29.1%-34.7%) | 38.7% (35.8%-41.6%) | 12.7% (10.8%-14.8%) | 16.8% (14.6%-19.1%) |  |
| Master degree (MSc, MA, etc) | 122 | 24.8% (17.1%-32.9%) | 47.2% (37.7%-56.1%) | 12.2% (6.7%-18.7%) | 15.9% (10.3%-23.9%) |  |
| Doctoral degree (PhD, EdD, etc.) | 38 | 31.1% (17.3%-48.4%) | 49.4% (33.3%-66.7%) | 9.3% (2.7%-23%) | 10.1% (2.7%-23%) |  |
| Other | 273 | 49.4% (44.3%-54.6%) | 33.7% (29%-38.7%) | 10.4% (7.7%-14%) | 6.5% (4.2%-9.2%) |  |
| Missing | 7 | 28.6% (7.7%-71.4%) | 42.9% (16.7%-83.3%) | 14.3% (1.9%-55.8%) | 14.3% (1.9%-55.8%) |  |

**Supplementary table 6.** Weighted relative frequency (95%CI) of conflicting responses to statements about identity versus setting, role and future, by association membership

|  | **n** | **Concordant: Traditional & Alternative** | **Discordant: Progressive & Alternative** | **Discordant: Traditional & Mainstream** | **Concordant: Progressive & Mainstream** | **Chi-Square Tests  Cramér's V** |
| --- | --- | --- | --- | --- | --- | --- |
| **Internal conflict: Identity vs. Setting (Training)** | | | | | | |
| No | 1252 | 21.1% (18.8%-23.6%) | 10.5% (8.9%-12.4%) | 27.9% (25.4%-30.6%) | 40.5% (37.6%-43.3%) | X^2^=19.85, df=6, P=0.003  Cramér's V=0.065 |
| Yes | 1057 | 18.7% (16.6%-21%) | 9.7% (8.1%-11.4%) | 22.5% (20.2%-25%) | 49.1% (46.3%-52%) |  |
| **Internal conflict: Identity vs. Setting (Primary care)** | | | | | | |
| No | 1250 | 4.8% (3.7%-6.2%) | 7.7% (6.3%-9.4%) | 44.2% (41.4%-47.1%) | 43.2% (40.4%-46.2%) | X^2^=16.729, df=6, P=0.01  Cramér's V=0.06 |
| Yes | 1054 | 3.7% (2.8%-4.9%) | 7.5% (6.1%-9.1%) | 37.6% (34.8%-40.4%) | 51.1% (48.3%-54%) |  |
| **Internal conflict: Identity vs. Role (Adjustment)** | | | | | | |
| No | 1251 | 14.7% (12.7%-16.8%) | 1.9% (1.3%-2.9%) | 34.4% (31.6%-37.1%) | 49% (46.1%-51.9%) | X^2^=17.736, df=6, P=0.007  Cramér's V=0.062 |
| Yes | 1061 | 14% (12.1%-16.1%) | 1.6% (1.1%-2.5%) | 27.3% (24.8%-29.9%) | 57.1% (54.3%-59.9%) |  |
| **Internal conflict: Identity vs. Role (Medication)** | | | | | | |
| No | 1248 | 42.4% (39.6%-45.3%) | 31.3% (28.6%-34%) | 6.7% (5.4%-8.3%) | 19.6% (17.3%-22%) | X^2^=18.894, df=6, P=0.004  Cramér's V=0.064 |
| Yes | 1061 | 36.7% (33.9%-39.4%) | 37.5% (34.8%-40.3%) | 4.6% (3.5%-5.9%) | 21.2% (18.9%-23.6%) |  |
| **Internal conflict: Identity vs. Future (Theory)** | | | | | | |
| No | 1254 | 8.2% (6.7%-9.9%) | 2.5% (1.7%-3.5%) | 40.9% (38.1%-43.8%) | 48.4% (45.5%-51.3%) | X^2^=16.886, df=6, P=0.01  Cramér's V=0.06 |
| Yes | 1058 | 6.1% (4.8%-7.6%) | 2.2% (1.5%-3.2%) | 35.2% (32.5%-38%) | 56.5% (53.7%-59.4%) |  |
| **Internal conflict: Identity vs. Future (Subgroups)** | | | | | | |
| No | 1251 | 35.6% (32.8%-38.4%) | 36.8% (34%-39.6%) | 13.5% (11.6%-15.5%) | 14.1% (12.2%-16.3%) | X^2^=22.137, df=6, P=0.001  Cramér's V=0.069 |
| Yes | 1061 | 31.6% (29.1%-34.4%) | 42.9% (40.1%-45.7%) | 9.6% (8%-11.4%) | 15.8% (13.8%-18%) |  |

**Supplementary table 7**. Weighted relative frequency (95%CI) of conflicting responses to statements about identity-2 versus setting, role and future

|  | **Concordant: Traditional & Alternative** | **Discordant: Progressive & Alternative** | **Discordant: Traditional & Mainstream** | **Concordant: Progressive & Mainstream** |
| --- | --- | --- | --- | --- |
| **Identity (Traditional or Progressive)** | 2. Contemporary and evolving scientific evidence is more important than traditional chiropractic principles | | | |
| **vs. Setting (Alternative or Mainstream)** |  |  |  |  |
| 1. Inclusion of clinical chiropractic training internships and post-graduate positions in integrative medical settings are important to the progression of the chiropractic profession [n=2366] | 19.1% (17.6%-20.7%) | 10.7% (9.5%-12%) | 25.3% (23.6%-27.1%) | 44.9% (42.9%-46.9%) |
| 2. Chiropractic providers should maintain its primary health care (direct access) status [n=2363] | 5.5% (4.6%-6.5%) | 6.4% (5.4%-7.4%) | 38.9% (37%-40.9%) | 49.1% (47.1%-51.2%) |
| **vs. Role (Alternative or Mainstream)** |  |  |  |  |
| 1. Chiropractic intervention should consist of chiropractic adjustment only [n=2372] | 11.1% (9.9%-12.4%) | 4.7% (3.9%-5.6%) | 33.2% (31.3%-35.1%) | 50.9% (48.9%-53%) |
| 2. The chiropractic profession should expand its scope of practice to include prescription of medication, with appropriate advanced training [n=2368] | 37.2% (35.3%-39.2%) | 36.3% (34.4%-38.3%) | 7.1% (6.1%-8.2%) | 19.3% (17.8%-20.9%) |
| **vs. Future (Alternative or Mainstream)** |  |  |  |  |
| 1. It is appropriate to allow for chiropractic theories to be updated and enhanced through the application and integration of current scientific advancements [n=2370] | 8.2% (7.1%-9.3%) | 1.5% (1%-2%) | 36.2% (34.3%-38.2%) | 54.1% (52.1%-56.1%) |
| 2. It is appropriate for the chiropractic profession to distinguish and promote two separate subgroups of intervention. 1) Providing manual and other non-drug procedures 2) Providing subluxation correction only [n=2370] | 34.7% (32.8%-36.7%) | 38.9% (37%-40.9%) | 9.7% (8.6%-10.9%) | 16.7% (15.2%-18.2%) |
